# Aortic Stiffness Is Related to the Ischemic Brain Injury Biomarker N-Methyl-D-aspartate Receptor Antibody Levels in Aortic Valve Replacement

**DOI:** 10.1155/2014/970793

**Published:** 2014-06-26

**Authors:** Emaddin Kidher, Vanash M. Patel, Petros Nihoyannopoulos, Jon R. Anderson, Andrew Chukwuemeka, Darrel P. Francis, Hutan Ashrafian, Thanos Athanasiou

**Affiliations:** ^1^Department of Surgery and Cancer, Imperial College London, 10th Floor, QEQM Wing, St Mary's Campus, London W2 1NY, UK; ^2^Departments of Cardiac Surgery and Cardiology, Imperial College Healthcare NHS Trust, London W12 0HS, UK; ^3^International Centre for Circulatory Health, Imperial College Healthcare NHS Trust, London W2 1LA, UK

## Abstract

*Background*. Aortic stiffness changes the flow pattern of circulating blood causing microvascular damage to different end-organ tissues, such as brain cells. The relationship between aortic stiffness measured by pulse wave velocity (PWV) and serum ischemic brain injury biomarker N-methyl-D-aspartate receptor antibody (NR2Ab) levels in aortic valve replacement has not been assessed. *Methods*. Patients undergoing aortic valve replacement (AVR) for aortic stenosis (AS) had their PWV and NR2Ab serum levels measured preoperatively. We analyzed PWV and NR2Ab in two ways: (1) as continuous variables using the actual value and (2) as dichotomous variables (PWV-norm and PWV-high groups) and (NR2Ab-low and NR2Ab-high groups). *Results*. Fifty-six patients (71 ± 8.4 years) were included in this study. The NR2Ab level (ng/mL) was significantly higher in the PWV-high group (*n* = 21) than in PWV-norm group (*n* = 35; median 1.8 ± 1.2 versus 1.2 ± 0.7, resp., *P* = 0.003). NR2Ab level was positively associated with PWV and negatively associated with male gender. Multiple regression revealed PWV independently related to NR2Ab level, and PWV cut-off was associated with a 7.23 times increase in the likelihood of having high NR2Ab (>1.8 ng/mL). *Conclusion*. Higher PWV in patients with surgical aortic stenosis is associated with higher levels of the ischemic brain biomarker NR2Ab.

## 1. Introduction

Aortic stiffness is an emerging risk factor for cardiovascular disease and it is related to age [1]. Aortic stiffness assessed by pulse wave velocity (PWV) is an independent predictor of cardiovascular (CV) and all-cause mortality in hypertension [2], diabetes mellitus [3], and end stage renal disease [4]. Carotid-femoral PWV is considered the gold standard for measuring arterial stiffness [1]. In 2010, the “Reference Values for Arterial Stiffness Collaboration” published the reference and normal values for PWV based on European population (eight countries) of more than 16 thousands subjects [1]. These reference values can be employed to investigate the relationship between arterial stiffness and other end points in different populations.

Vascular autoregulation maintains a relatively stable brain blood flow despite blood pressure changes. In addition, regional increases in brain activity cause regional surges in blood demand and influences regional increases in blood flow, a process known as functional hyperemia [5]. Efficient global brain circulation, autoregulation, and functional hyperemia depend not only on blood volume and blood pressure but also critically on intact, healthy, and reactive cerebral blood vessels [5].

In a longitudinal study of 1,715 essential hypertensive patients with a mean follow-up of 7.9 years, PWV independently predicted fatal stroke after full adjustment for classic cardiovascular risk factors, including age, cholesterol, diabetes, smoking, mean blood pressure, and pulse pressure [6]. Furthermore, Okuyama et al. found that aortic stiffness is an independent predictor of stroke recurrence after a mean follow-up period of 459 days for 201 stroke patients [7]. Moreover, there is an association between aortic PWV and intracranial large artery disease in patients with history of ischemic stroke [8]. The mechanism of progressive neurological deficit in patients with acute deep subcortical infarction is unknown; however, Saji et al. have recently hypothesized that PWV may be part of such a mechanism, as they found that PWV was independently associated with progressive neurological deficit in these patients [9]. As the relationship between aortic stiffness and cerebrovascular outcome was established, additional studies on the mechanisms and mediators of this relationship using advanced imaging technology were more prevalent. PWV is independently associated with the manifestations of cerebral small vessel disease, such as silent brain infarction and white matter disease, which are risk factors for stroke [10–12]. Using advanced magnetic resonance imaging (MRI) arterial spin labeling (ASL) technique, Tarumi et al. found a significant inverse relationship between aortic stiffness and cerebral perfusion of frontal white matter [13] and the occipitoparietal region [14]. The damaging effect of vascular risk factors including central arterial stiffness on the brain tissue by different mechanisms such as chronic hypoperfusion has been investigated widely using imaging technologies which are expensive and have practical limitations [15, 16]. Therefore, identifying a practical and cheaper biomarker linking vascular pathology to brain injury would be clinically vital.

Ischemic brain biomarkers have been extensively studied for decades to identify a reliable and practical biomarker equivalent to the cardiac troponins. There are about 70 ischemic brain injury biomarkers which have been studied over the last four decades; none of them is currently used in clinical practice for the diagnosis of brain ischemia [17–19]. Some of these biomarkers have been identified as potential candidates for future studies involving ischemic injuries, including NR2Ab, S-100, tau protein, NSE, brain natriuretic peptide, and CRP [17, 18, 20–24]. Our literature search could not find any study investigating brain biomarkers in chronic cerebral ischemia. Therefore, the predictive value of such biomarkers and their association with other potential risk factors of chronic brain ischemia, such as aortic stiffness, is an area of research interest.

Death or ischemia of neural cells causes degradation of subunits (NR2) of the NMDA receptor, and the proteolytic fragments start to circulate in the bloodstream. This in turn stimulates the immune system to produce antibodies against these NR2 fragments (NR2Ab), which can be assayed in blood samples [25]. Adult patients with acute or recent ischemic stroke have elevated blood levels of NR2Ab that correlate with the amount of brain damage or the presence of recent stroke in comparison to controls. Test sensitivity and specificity for diagnosing ischemic stroke within 3 hours of symptom onset were 100% and 89% for cut-off of 1.8 ng/mL and 97% and 98% for cut-off point of 2.0 ng/mL [26, 27]. Bokesch et al. found that preoperative serum concentrations of NR2Ab are predictive of severe neurological adverse events after cardiac surgery in high-risk adult and that patients with NR2Ab level ≥2.0 ng/mL (selected based on the above study [26]) preoperatively were nearly 18 times more likely to develop neurological event postoperatively than patients with NR2Ab <2.0 ng/mL [20]. Physiologically, the production of these antibodies may take time, and they last in the blood for many months after ischemia, making the clinical applications of this test not limited to acute setting [17, 19, 28]. This supports the hypothesis of investigating NR2Ab level in more chronic pathologies such as arterial stiffness. NR2Ab is a potential brain injury biomarker, but its relationship with aortic stiffness has never been investigated.

The relationship between aortic stiffness and cardiovascular and cerebrovascular adverse outcome has been established. Therefore, identifying a biomarker (such as NR2AB) linking the vascular cause (such as aortic stiffness) to end-organ damage (such as the brain) is of clinical importance.

## 2. Materials and Methods

### 2.1. Patient Population and Overview

Ethical approval (11/H0709/3) and participant informed consent were obtained prior to the study. From June 2010 to August 2012, patients planning to undergo AVR for severe aortic stenosis were eligible. Exclusion criteria included (1) aortic dissection; (2) severe aortic regurgitation; (3) thoracic aorta (more than just the root) or abdominal aortic aneurysm; (4) Marfan's syndrome; (5) a history of stroke or transient ischemic attack (TIA) in the last year.

Carotid-femoral PWV was used to assess aortic stiffness. To measure NR2Ab, blood samples were collected from each patient preoperatively [20, 26]. Based on patients' preoperative PWV measurement and the published European normal values of PWV for the matched age group [1], patients were divided into two groups:

- PWV-norm group: with PWV equal to or below normal reference value for their respective age group.
- PWV-high group: with PWV above the normal reference value for their respective age group.

### 2.2. Pulse Wave Velocity Measurement Protocol

Abstinence from tea, coffee, and tobacco for at least 2 hours prior to measurement was ensured. Patients were asked to rest for at least 10 minutes in a quiet, temperature-controlled room (22–25 degree Celsius) before the hemodynamic measurements were taken. Automated digital sphygmomanometer (Criticare system, Model 506N3, Waukesha, USA) was used to measure brachial blood pressure (BP) and heart rate. Separated by the PWV measurements, two readings of BP were taken, and the mean was used for analysis. Automatic applanation tonometry system (the SphygmoCor Vx system, AtCor Medical, Australia) was employed to measure aortic PWV (carotid-femoral PWV). While resting in a supine position, ECG-gated pulse waveforms were obtained sequentially from the common carotid and then femoral arteries by application of applanation tonometry. Propagation time of the pulse wave was measured from the foot of the carotid waveform to that of the femoral waveform referenced to the R-wave on the recorded ECG. The travelling distance of the waveform was measured in millimeters externally by subtracting the distance between the suprasternal notch and carotid site from the distance between the suprasternal notch and femoral site. The system software of the SphygmoCor device calculated PWV automatically in meters per second (m/s) by dividing the travelled distance by the propagation time. Three to five readings of PWV were obtained per patient; PWV was determined by averaging the measurements that met the quality control parameters (as set by SphygmoCor Clinical User Manual).

### 2.3. NR2Ab Biomarker

Blood samples were collected 18–24 h prior to surgery from 52 patients out of 56 patients who had their PWV measured. Blood samples were centrifuged immediately with 3000 rpm at 20°C for 10 minutes, and then serum samples were separated and frozen at −80°C. Frozen samples were processed with ELISA technique using Gold Dot NR2 Antibody Test (CIS Biotech, Inc., Atlanta, GA). This was done according to the manufacturer's procedure and as previously published [27]. In brief, frozen samples were thawed at −8°C. Serum sample was diluted 1 : 50 (20 *μ*L of serum sample + 980 *μ*L of working buffer) into 3 mL tubes. Microplates were washed with working buffer for 5 minutes at 37°C on a shaker. We then added 100 *μ*L of calibrators, negative controls, positive controls, and prepared diluted sera to microplates coated with NR2 peptide and incubated the plates for 30 minutes at 37°C on a shaker. After incubation, the microplates were washed with buffer, and 100 *μ*L (1 : 1000 dilution) of A-HRP (antihuman horseradish peroxidise) was added to the microplates and incubated for 30 minutes at 37°C on a shaker to structure the immunocomplex. The microplates were then washed with working buffer and distilled water and 100 *μ*L of TMB (tetramethylbenzidine) substrate was added and incubated in darkness at room temperature for 10 minutes to accomplish colour development. Colour reaction stopped by adding Stop Reagent (100 *μ*L) and gentle shaking for 30 seconds. The optic density was measured within 10 minutes at 450 nm/630 nm dual wave microplate reader. The NR2A antibodies in serum were measured by using a standard calibration curve of the absorbance obtained for each calibrator *X*-axis versus the NR2 antibody concentration in ng/mL on the *Y*-axis.

### 2.4. Statistical Analysis

Statistical analysis was conducted using IBM SPSS 20.0 software package (IBM Corporation, Armonk, NY, USA). Patient characteristics and results are expressed as means ± standard deviation, except for NR2Ab, which is expressed as median ± standard deviation, for continuous variable and as frequencies for categorical variables. *P* values <0.05 were considered statistically significant. Normality testing was carried out on all variables studied. For the purpose of the regression analysis, nonnormally distributed NR2Ab levels were log-transformed.

PWV was analyzed as a continuous variable to verify our hypothesis. In addition, it was also analyzed as a dichotomous variable (PWV-norm group versus PWV-high group). NR2Ab was analyzed as a continuous variable by using the actual serum level to verify our hypothesis. Additional analysis as a dichotomous variable was performed using the median value of the NR2Ab level of the PWV-high group (1.8 ng/mL) as a cut-off point to classify patients into NR2Ab-low (<1.8 ng/mL) and NR2Ab-high (≥1.8 ng/mL) groups; this cut-off was previously tested [26]. Comparative analysis between the two PWV groups (PWV-norm and PWV-high) and the two NR2Ab groups (NR2Ab-low and NR2Ab-high) was carried out using an independent samples* t*-test or nonparametric equivalent (Mann-Whitney *U* test) for continuous variables, and Pearson Chi-square or Fisher's exact tests were carried out for categorical variables. Correlation analysis between variables was conducted using Spearman's rank-order correlation and point biserial correlation (for dichotomous variables), while Phi test was used between two dichotomous variables (2 × 2). Simple regression (linear or logistic) between end points and other variables were used to identify potential predictors. All variables significant by simple regression or correlation, in addition to age and gender, were then included in two models of multiple regression analysis (enter method): (1) Model 1, including PWV as a continuous predictor; and (2) Model 2, including PWV cut-off as a dichotomous predictor.

## 3. Results

### 3.1. Descriptive Results

Fifty-six patients (16 females) with a mean age of 71 ± 8.4 years were recruited for this study. No mortality or severe neurological event (stoke or TIA) was recorded after a mean follow-up period of 409 ± 159 days postoperatively (post-AVR).  Table 1 summarizes the demographic and clinical data of the patients and the correlation of these variables with NR2Ab level and the NR2Ab 1.8 ng/mL cut-off. NR2Ab was significantly associated with aortic stiffness (PWV), while the NR2Ab 1.8 ng/mL cut-off, in addition to PWV, was negatively associated with male gender.

### 3.2. NR2Ab and Aortic Stiffness

The overall mean PWV value was 9.3 ± 2.2 m/s and, as expected, it was significantly related to different age groups (*P* = 0.001, analysis of variance [ANOVA]) but not gender (*P* = 0.34). Thirty-five (62.5%) patients were classified in the PWV-norm group and 21 patients (37.5%) were in the PWV-high group. There was no significant difference between the two groups of PWV with respect to age, gender, classical hemodynamic measurements, aortic valve mean gradient, aortic valve peak gradient and aortic valve area, estimated IQ,, and other clinical characteristics (data is not shown).

The NR2Ab level (ng/mL) was significantly higher in the PWV-high group than in the PWV-norm group (median 1.8 ± 1.2 versus 1.2 ± 0.7, resp., *P* = 0.003), and PWV (m/s) was significantly higher in the NR2Ab-high group (*n* = 17) than in the NR2Ab-low group (*n* = 35; mean 10.9 ± 2.1 versus 9.1 ± 2.2, *P* = 0.05).  Table 1 demonstrates the significant correlations between NR2Ab and PWV value (*r* = 0.28, *P* = 0.05), NR2Ab and PWV cut-off (*r* = 0.42, *P* = 0.002), NR2Ab 1.8 ng/mL cut-off and PWV value (*r* = 0.27, *P* = 0.05), and NR2Ab 1.8 ng/mL cut-off and PWV cut-off (Phi = 0.43, *P* = 0.002).

To confirm the findings of the correlation analysis, simple linear regression between NR2Ab and other variables (age, gender, mean arterial pressure, body mass index, smoking diabetes mellitus, ejection fraction, aortic valve peak gradient, aortic valve mean gradient, aortic valve area, cholesterol, triglycerides, and PWV) was performed to identify potential predictors of NR2Ab level (Table 2). In this simple linear regression analysis, the relationship between NR2Ab (log transformation) was significantly related only to PWV value (continuous), PWV cut-off (dichotomous), and gender. Variables with significant linear regression or correlation, in addition to age, were included in a multiple regression (enter method) analysis (Table 2). Model 1 of the multiple linear regression (includes PWV, age, and gender) statistically significantly predicts NR2Ab (*F* = 3.33, *P* = 0.03), and 17.2% of variance in NR2Ab can be explained by changes in these variables. PWV was independently related to NR2Ab level (beta = 0.37; *P* = 0.022). Model 2 of the multiple linear regression (including PWV cut-off, age, and gender) statistically significantly predicts NR2Ab level (*F* = 4.13, *P* = 0.01), and 20.5% of variance in NR2Ab can be explained by changes in these variables. The PWV cut-off was independently related to NR2Ab level (beta = 0.37; *P* = 0.008).

Additional analysis was performed to ascertain the effects of PWV on the likelihood of patients being in the NR2Ab-high group (≥1.8 ng/mL). First, simple logistic regression was conducted to confirm the findings from the correlation analysis; only PWV cut-off and gender were statistically significant, while the PWV value was clinically significant (Table 3). Therefore, only PWV (the predictor of interest) and age and gender (classical variables) were included in the multiple logistic models. In Model 1 of the multiple logistic regression (Table 3), though the entire model was statistically significant (*P* = 0.017) and was explained to be 24.7% (Nagelkerke *R* ^2^) of the NR2Ab variance, the PWV value itself was not a significant predictor, while gender was the only significant predictor, of which male gender was protective (odds ratio [OR] below 1). Model 2, using PWV as a dichotomous variable (PWV-norm versus PWV-high), was even more significant than Model 1. The logistic regression model was statistically significant (*P* = 0.002). The model explained 35.5% (Nagelkerke *R* ^2^) of the variance in NR2Ab and correctly classified 73.1% of cases, the positive predictive value was 66.6%, and the negative predictive value was 74.4%. In this model, only PWV cut-off was statistically significant, as changing the status of PWV from normal to high was associated with a 7.23 times increased likelihood of having high NR2Ab (>1.8 ng/mL; Table 3).

## 4. Discussion

Most of the previous studies focused on the predictive value of the NR2Ab in acute ischemia (stoke and TIA) [26, 27]; however, little has been done to explore the predictive value in more chronic cerebrovascular pathologies such as arterial stiffness. In our 52 patients, NR2Ab levels were not related to age. Conversely, male gender was related to lower NR2Ab level. Furthermore, our results showed that only two variables, gender and PWV, were associated with NR2Ab level; male gender had a negative association, while PWV (aortic stiffness) had a positive association. To test the objective of whether PWV is related to NR2Ab biomarker level, correlation and multiple regression analyses were performed. These analyses revealed that PWV value was significantly related to and independently predicted NR2Ab but not the NR2Ab 1.8 ng/mL cut-off. Grouping patients according to their PWV into PWV-norm and PWV-high (PWV cut-off) increased the significance of the relationship with NR2Ab, PWV cut-off was an independent predictor of NR2Ab level, and it was associated with a 7.23 times increased likelihood of having high NR2Ab > 1.8 ng/mL.

It is known that high PWV is significantly associated with cerebral small vessel disease [29–31] and NR2Ab is a potential ischemic brain injury biomarker [25–27]. The positive relationship between NR2Ab and PWV may be explained by the hemodynamic ischemic effect of arterial stiffness on brain tissue and subsequent release of proteolytic fragments of NR2 subunits of the NMDA receptors and the formation of NR2Ab over time. This does not undermine the importance of NR2Ab in acute ischemia, particularly if serum levels are proportional to the size and severity of the ischemia. For example, it was previously reported that the mean NR2A/2B antibody level (ng/mL) in patients with ischemic stroke was 5.01 ± 1.23 (range, 3.24–7.21), in patients with TIA it was 4.02 ± 2.04 (range, 2.71–7.23), and in patients with hypertension/atherosclerosis it was 1.72 ± 0.23, while in controls it was only 1.49 ± 0.22 (range, 1.02–1.98) [26]. Our results are similar to the values of the atherosclerosis and controls groups (median 1.8 ± 1.2 versus 1.2 ± 0.7 for the PWV-high versus PWV-norm groups, resp.) as no patient developed stroke or had a TIA.

There was no correlation between AS parameters, such as AVA, AVMG, or AVPG, and NR2Ab level (Table 1). The absence of such a relationship may indicate that AS has no chronic ischemic effect on brain tissue. However, this conclusion would be more concrete if we measured NR2Ab levels postoperatively to determine if replacing a diseased valve (AVR) has any effect on NR2Ab levels. To the best of our knowledge, this is the first study to investigate and establish a relationship between aortic stiffness (PWV) and NR2Ab biomarker levels.

## 5. Limitations

NR2Ab level was only measured preoperatively; it may be useful to measure sequential levels postoperatively to determine whether there is a change in NR2Ab level following AVR. The effect of potential confounders, such as diabetes, hypertension, and dyslipidemia, on the relationship between aortic stiffness and NR2Ab requires a much larger sample size to be adequately evaluated. However, the aim of this study was to provide explorative preliminary data that can be used to direct future studies.

## 6. Conclusions and Future Clinical Prospects

Higher PWV values in patients with surgical aortic stenosis are associated with higher levels of the ischemic brain biomarker NR2Ab.

As for any predictor of disease condition, early identification of patients at risk will facilitate regular monitoring, risk stratification, and future planning and consideration of potential therapies, if available. PWV is a predictor of adverse cardiovascular and cerebrovascular outcomes, while NR2Ab is a potential pathophysiological predictor of adverse cerebrovascular outcomes. The association between two predictors of common or different outcomes, for example, between diabetes and hypertension or in our case between PWV and NR2Ab, provides a better understanding of the disease development process, facilitates the development of future risk stratification models, and renders their clinical use, to some degree, interchangeable.

This study offers a significant step forward in our knowledge pertaining to end-organ damage in the presence of increased aortic stiffness. Future cross-sectional studies with larger sample sizes to adjust for potential confounders, such as hypertension, diabetes, and dyslipidemia, are required to establish or reject this relationship. Establishing such a relationship in cross-sectional studies will warrant the need for longitudinal studies to assess the predictive value of NR2Ab level for clinical outcomes across different groups of PWV values. Ultimately, this will move us a step closer to discovering new clinical (PWV) and biochemical (NR2Ab) predictors of neurocognitive outcomes, in addition to the traditional risk factors currently in use.

## Figures and Tables

**Table 1:** Demographic and clinical characteristics and their correlation with NR2Ab level and NR2Ab 1.8 ng/mL cut-off.

Variables	Total (*n* = 56)	NR2Ab level ^a^ *r*	NR2Ab 1.8 ng/mL cut-off ^a^ *r*
PWV (m/s)	9.3 ± 2.2	**0.28** ∗	**0.27** ∗
PWV cut-off	**—**	**0.42** ∗∗	**0.43** ∗∗
NR2Ab 1.8 ng/mL cut-off	**—**	**0.74** ∗∗	1.00
Age (years)	71 ± 8.4	−0.08	0.01
Gender (male)	40 (71.4%)	−0.26	−**0.32**∗
DM [*n* (%)]	8 (14.3%)	0.14	0.09
Smoking [*n* (%)]	2 (3.6%)	−0.08	0.02
BMI (kg/m^2^)	27.2 (4.2)	−0.21	−0.23
SBP (mmHg)	136 ± 24	−0.03	0.02
DBP (mmHg)	76 ± 11	−0.22	−0.24
PP (mmHg)	62 ± 15	0.11	0.21
MAP (mmHg)	97 ± 12	−0.11	−0.15
Cholesterol (mmol/L)	4.5 ± 1.2	0.23	0.22
Hypertension [*n* (%)]	38 (67.9%)	0.18	0.14
Statin treatment [*n* (%)]	38 (67.9%)	0.10	0.02
PVD [*n* (%)]	2 (3.6%)	0.12	0.25
EuroSCORE (logistic)	5.5 ± 4.3	0.04	0.18
AVA (cm^2^)	0.73 ± 0.2	−0.09	−0.10
AVMG (mmHg)	48 ± 13	0.00	−0.06
AVPG (mmHg)	82 ± 24	0.14	0.01
EF	59 ± 15	0.22	0.06

*Correlation is significant at the 0.05 level (2-tailed); **correlation is significant at the 0.01 level (2-tailed). ^a^Correlation coefficient, bold values indicate statistical significance. Abbreviations: AVA, aortic valve area; AVMG, aortic valve mean gradient; AVPG, aortic valve peak gradient; BMI, body mass index; DBP, diastolic blood pressure; DM, diabetes mellitus; EF, Ejection fraction; MAP, mean arterial blood pressure; NR2Ab, N-methyl-D-aspartate (NMDA) receptor antibodies; PP, pulse pressure; PVD, peripheral vascular disease; PWV, pulse wave velocity; SBP, systolic blood pressure.

**Table 2:** Simple and multiple linear regression analysis to identify variables that independently predict NR2Ab level.

Predictors	NR2Ab level beta (*P* value)
Simple linear regression	
PWV value	**0.27 (0.05)**
PWV cut-off	**0.39 (<0.01)**
Age	−0.05 (0.71)
Gender (male)	−**0.27 (0.05)**
DM	0.08 (0.57)
Smoking	−0.12 (0.38)
BMI (kg/m^2^)	−0.2 (0.15)
MAP (mmHg)	−0.16 (0.26)
Cholesterol (mmol/L)	0.09 (0.67)
EuroSCORE (logistic)	0.07 (0.49)
AVA (cm^2^)	−0.09 (0.55)
AVMG (mmHg)	−0.01 (0.92)
AVPG (mmHg)	−0.06 (0.66)
EF	0.03 (0.83)

Multiple linear regression (Model 1)	
PWV value	**0.37 (0.02)**
Age	−0.25 (0.12)
Gender (male)	−0.21 (0.12)

Multiple linear regression (Model 2)	
PWV cut-off	**0.37 (<0.01)**
Age	−0.09 (0.46)
Gender (male)	−0.21 (0.11)

Values are shown as standardized beta coefficient (*P* value); bold values indicate statistical significance. Abbreviations: AVA, aortic valve area; AVMG, aortic valve mean gradient; AVPG, aortic valve peak gradient; BMI, body mass index; DM, diabetes mellitus; EF, Ejection fraction; MAP, mean arterial blood pressure; NR2Ab, N-methyl-D-aspartate (NMDA) receptor antibodies; PWV, pulse wave velocity.

**Table 3:** Simple and multiple logistic regression analysis to identify variables that independently predict patients with high NR2Ab (NR2Ab 1.8 ng/mL cut-off).

Predictors	NR2Ab 1.8 ng/mL cut-off	
	OR (95%CI)	*P*-value
Simple logistic regression		
PWV value	1.29 (0.97–1.71)	0.08
PWV cut-off	**6.93 (1.91–25.17)**	**<0.01**
Age	1.0 (0.93–1.01)	0.96
Gender (male)	**0.18 (0.05–0.67)**	**0.01**
DM	1.63 (0.33–8.01)	0.54
Smoking	0.94 (0.33–2.65)	0.92
BMI (kg/m^2^)	0.88 (0.79–1.02)	0.09
MAP (mmHg)	0.97 (0.92–1.02)	0.31
Cholesterol (mmol/L)	1.63 (0.84–3.19)	0.14
EuroSCORE (Logistic)	1.09 (0.95–1.25)	0.21
AVA (cm^2^)	0.35 (0.01–6.98)	0.49
AVMG (mmHg)	0.99 (0.64–1.03)	0.69
AVPG (mmHg)	1.0 (0.97–1.02)	0.96
EF	1.0 (0.96–1.05)	0.72

Multiple logistic regression (Model 1)		
PWV value	1.4 (0.94–2.06)	0.09
Age	0.95 (0.86–1.05)	0.32
Gender (male)	**0.21 (0.05–0.79)**	**0.02**

Multiple logistic regression (Model 2)		
PWV cut-off	**7.23 (1.74–30.1)**	**<0.01**
Age	0.98 (0.91–1.06)	0.66
Gender (male)	**0.19 (0.04–0.8)**	**0.02**

Values are shown as odd ratio (OR) and *P* value; bold values indicate statistical significance. Abbreviations: AVA, aortic valve area; AVMG, aortic valve mean gradient; AVPG, aortic valve peak gradient; BMI, body mass index; DM, diabetes mellitus; EF, Ejection fraction; MAP, mean arterial blood pressure; NR2Ab, N-methyl-D-aspartate (NMDA) receptor antibodies; PWV, pulse wave velocity.
